# Increased 24 h Urinary Cadmium Excretion Following Intravenous Ca-DTPA: A Retrospective Paired Analysis

**DOI:** 10.3390/jox16060197

**Published:** 2026-10-16

**Authors:** Richard C. Semelka, Miguel Ramalho

**Affiliations:** 1 Richard Semelka Consulting, PLLC, Chapel Hill, NC 27516, USA; richardsemelka@gmail.com; 2 Department of Radiology, Hospital da Luz, 1500-650 Lisbon, Portugal

**Keywords:** cadmium, urinary excretion, calcium-DTPA, chelation, retrospective paired analysis

## Abstract

Cadmium is a widespread environmental toxicant associated particularly with renal tubular injury and skeletal disease. Human evidence regarding chelation-enhanced cadmium elimination is limited. This retrospective paired analysis evaluated changes in 24 h urinary cadmium excretion following intravenous calcium diethylenetriaminepentaacetate (Ca-DTPA) in 60 patients treated primarily for suspected gadolinium-related conditions. The cohort included 30 women and 30 men, aged 21–79 years (mean, 49 years). Mean urinary cadmium excretion increased from 0.32 to 1.31 µg/24 h following Ca-DTPA administration, corresponding to a mean paired increase of 0.99 µg/24 h (95% CI, 0.75–1.23 µg/24 h). The ratio of the post-treatment to pretreatment group means was 4.09, while the median individual post/pre ratio was approximately 3.7 (IQR, 2.4–5.8; range, 1.2–9.5). All 60 participants showed increased urinary cadmium excretion (Wilcoxon signed-rank *p* < 0.001; two-sided exact sign-test *p* = 1.73 × 10^−18^). These findings demonstrate increased urinary cadmium excretion following Ca-DTPA under the conditions studied, but do not establish a clinically meaningful reduction in total-body cadmium burden, treatment safety, or clinical benefit.

## 1. Introduction

Cadmium is a persistent environmental contaminant with well-established toxic effects in humans, particularly on the kidneys and bone [1,2,3,4]. Long-term exposure has been associated with renal tubular damage, impaired bone mineralization, osteoporosis, and bone pain [1,2,3,4,5]. Cadmium exposure may occur through diet, tobacco smoke, occupational settings, and contamination of water or soil. The consequences of high environmental exposure were most clearly illustrated by Itai Itai disease in Japan, in which chronic cadmium exposure was associated with renal dysfunction, disturbances in mineral metabolism, osteomalacia, and severe skeletal pain [4].

Three broad strategies are relevant to toxic-metal management: avoidance of continued exposure, support of endogenous detoxification and elimination, and active removal with a chelating agent. For cadmium, the literature has focused mainly on exposure prevention and treating organ complications. Human evidence on chelation for cadmium exposure or toxicity is limited and includes small studies and case reports; the optimal treatment as well as its long-term clinical benefit remain uncertain [5,6].

Tominaga et al. evaluated several DTPA formulations and routes in healthy male volunteers and demonstrated increased urinary cadmium excretion after intravenous DTPA [7]. Gil et al. documented increased urinary cadmium excretion during a CaNa2EDTA-plus-glutathione protocol in a patient with chronic cadmium intoxication [8]. By contrast, a placebo-controlled analysis of succimer in children with background cadmium exposure found no reduction in blood cadmium [9]. These studies used different chelators, populations, exposure settings, and biomarkers and therefore do not establish an optimal treatment for chronic cadmium toxicity.

Ca-DTPA is approved in the United States as a decorporation agent after known or suspected internal contamination with plutonium, americium, or curium [10]. It is not approved for cadmium poisoning or for gadolinium-related conditions. Guidance for emergency radionuclide decorporation concerns a different clinical setting [11].

In the present cohort, Ca-DTPA was administered primarily to patients with symptoms attributed clinically to previous gadolinium exposure rather than to patients recruited for cadmium poisoning [12,13,14]. This secondary analysis sought to characterize the within-person change in 24 h urinary cadmium excretion following a qualifying 1 g intravenous Ca-DTPA administration. It was not designed to determine Ca-DTPA efficacy for cadmium poisoning, the fraction of total-body cadmium removed, or effects on cadmium-associated symptoms or organ injury.

For the present study, Doctor’s Data generated a research extract specifically for cadmium. The file supplied for this analysis contained 24 h urinary cadmium results, along with the collection and demographic fields required for the paired analysis. This cadmium-focused extraction was intentional so that cadmium could be evaluated as a distinct endpoint.

## 2. Materials and Methods

### 2.1. Study Population

We included patients treated at a private medical clinic between January 2023 and January 2025 who had received intravenous Ca-DTPA for suspected gadolinium-related conditions and had urinary cadmium measurements available both before and after treatment. Patients were not selected because they had been diagnosed with cadmium poisoning. We recognize that patients referred to a specialized clinic may not represent the wider population, and that including only those with paired 24 h urine measurements may introduce further selection bias. These factors should be considered when interpreting the findings and limit how broadly the results can be applied.

The study included 60 patients, 30 women and 30 men, aged 21–79 years (mean age, 49 years). Only one qualifying Ca-DTPA treatment episode was included for each patient. Participant characteristics and the main treatment and urine collection details are summarized in Table 1.

### 2.2. Urine Collection and Cadmium Measurement

Twenty-four-hour urine collections were obtained in metal-free containers within 3 days before chelation and again beginning approximately 1 h after Ca-DTPA administration. The immediate post-chelation urine was not included because urine present in the bladder at the time of treatment would largely have been formed before administration of the chelating agent. We excluded that void because much of the bladder urine had formed before treatment, although we may also have omitted some early post-dose excretion. Accordingly, the endpoint is a subsequent 24 h collection beginning approximately 1 h after dosing, not the complete first 24 h after administration.

Samples were analyzed for 20 potentially toxic and essential metals at a commercial laboratory (Doctor’s Data, St. Charles, IL, USA) using dynamic reaction cell inductively coupled plasma–mass spectrometry (DRC-ICP-MS). Doctor’s Data measured urinary cadmium during routine clinical testing. For this study, Doctor’s Data supplied a cadmium-specific research extract containing total urinary cadmium together with collection volume, creatinine, demographic variables, treatment designation, and collection timing. We could not independently verify the completeness of each 24 h collection retrospectively; missed voids, inaccurate collection duration, contamination, or recording error therefore remain possible sources of measurement variability.

Urinary cadmium was measured as the total amount excreted over 24 h, expressed in µg/24 h, rather than as a concentration in a single urine sample or a value adjusted for creatinine. This approach reduces the influence of urine dilution, although it still depends on how accurately the 24 h collection was performed. It also does not tell us how much cadmium is stored in the body or how much of that total burden was removed.

We interpreted post-chelation urinary cadmium as a measure of cadmium mobilizable by Ca-DTPA under the conditions studied, not as a direct measure of total-body cadmium burden.

### 2.3. Chelation Protocol

All participants received 5 mL (1 g) of intravenous Ca-DTPA. We administered Ca-DTPA by manual intravenous injection, followed by a normal saline infusion.

Ca-DTPA is a multidentate aminopolycarboxylate ligand that can coordinate cadmium. In vivo decorporation depends not only on intrinsic metal-ligand affinity but also on physiological pH, competing ions and proteins, tissue distribution, kinetic accessibility of the retained metal, and renal elimination of the resulting complex. The present total-element measurements do not directly identify the chemical species of urinary cadmium.

The clinical protocol also included intravenous methylprednisolone, normal saline, and a short post-treatment corticosteroid and antihistamine regimen used to manage chelation-associated symptoms [13]. Because these were co-administered in an uncontrolled clinical setting, the study cannot isolate their independent contributions to urinary cadmium excretion.

### 2.4. Ethics

We retrospectively reviewed anonymized records from patients treated at a private medical clinic. All urine testing and Ca-DTPA treatments were part of routine clinical care. We added or changed nothing for this study, and we performed the analysis only after the clinical data had already been collected.

We analyzed anonymized data from routine clinical care. No identifiable patient information was included. Per local requirements and the clinic’s policy for this type of retrospective work, Institutional Review Board review was not required. The work followed the principles of the Declaration of Helsinki.

### 2.5. Statistical Analysis

We treated pre- and post-treatment cadmium measurements as paired observations. Descriptive reporting includes the pretreatment and post-treatment group means, the pretreatment range, and individual post/pre ratios summarized by median, interquartile range (IQR), and range. The ratio of the post-treatment group mean to the pretreatment group mean is reported separately and is not described as the arithmetic mean of individual fold changes.

We compared pre- and post-treatment values using a two-sided Wilcoxon signed-rank test. Statistical significance was defined as *p* < 0.05. Because all 60 participants showed an increase, we also calculated an exact two-sided sign test as a sensitivity analysis. The mean paired difference in urinary cadmium excretion and its corresponding 95% confidence interval were calculated from the individual pre- and post-treatment differences.

## 3. Results and Discussion

All 60 participants had detectable urinary cadmium before and after chelation, and every participant showed higher urinary cadmium excretion following Ca-DTPA administration. No participant had an unchanged or lower post-treatment value.

Pretreatment urinary cadmium ranged from 0.03 to 0.99 µg/24 h, with a mean of 0.32 µg/24 h. Following Ca-DTPA administration, mean urinary cadmium excretion increased to 1.31 µg/24 h, corresponding to a mean paired increase of 0.99 µg/24 h (95% CI, 0.75–1.23 µg/24 h). The post-treatment group mean was 4.09 times the pretreatment group mean (1.31/0.32). This is a ratio of group means, not the arithmetic mean of individual post/pre ratios. The median individual post/pre ratio was approximately 3.7 (IQR, 2.4–5.8), with a range of 1.2–9.5.

Post-treatment urinary cadmium was significantly higher than pretreatment urinary cadmium (Wilcoxon signed-rank test, *p* < 0.001). The exact two-sided sign test based on 60 increases among 60 paired observations was also significant (*p* = 1.73 × 10^−18^) (Table 2).

The increase in urinary cadmium excretion observed after Ca-DTPA suggests that the treatment may mobilize cadmium that is accessible to the chelator. However, we cannot determine where this cadmium came from, whether it was present in a particular chemical form, or how much of the body’s total cadmium burden was actually removed. Increased urinary excretion does not necessarily mean that cadmium stored in tissues has been significantly reduced, nor does it establish any clinical benefit.

Previous human studies provide some useful context, although they are not directly comparable with ours. Tominaga et al. reported increased urinary cadmium excretion after intravenous DTPA in healthy volunteers [7]. Gil et al. also observed increased urinary cadmium in a patient with chronic cadmium intoxication who received CaNa2EDTA together with glutathione [8]. On the other hand, a randomized study in children with background cadmium exposure found that succimer did not reduce blood cadmium levels [9]. These studies involved different populations, chelators, treatment schedules, and outcome measurements, making it difficult to draw conclusions about their relative effectiveness.

Animal studies have also shown that the timing of treatment can make a considerable difference. Following acute cadmium exposure, chelators such as DTPA, EDTA, and DMSA were generally more effective when given early. When treatment was delayed, urinary cadmium excretion could still increase, but the reduction in cadmium stored in tissues was much smaller [15,16,17,18]. This distinction is particularly relevant to our findings. An increase in urinary excretion does not necessarily indicate that cadmium retained inside cells has been removed, and results from acute exposure in animal models may not reflect what happens in humans with long-standing cadmium accumulation. Other animal studies have examined DTPA-based cadmium chelation and the influence of methionine as an adjunct [19,20].

The available human studies do not establish an accepted Ca-DTPA regimen for symptomatic cadmium toxicity. We did not recruit this cohort for cadmium-associated disease, and we did not measure cadmium-specific clinical outcomes. The study should therefore be interpreted as a mechanistic urinary-excretion analysis rather than a treatment-efficacy trial.

Chelator selection is a coordination-chemistry problem that also requires biological validation. Metal-ligand affinity is relevant, but in vivo performance depends on conditional stability at physiological pH, competition from endogenous ligands and metals, protein binding, tissue distribution, kinetic accessibility, and elimination of the resulting complex [21,22,23,24].

The present study contains no direct comparator chelator and therefore cannot rank Ca-DTPA against DMSA, EDTA, DMPS, or other agents. Speciation modeling by Jones et al. shows why isolated formation constants cannot predict removal of protein-bound cadmium in plasma [24]. The present data support only the observed association between Ca-DTPA administration and urinary cadmium excretion under the conditions studied.

Intracellular sequestration is another important limitation. Cadmium may bind to metallothionein and other intracellular ligands in the liver, kidney, and other tissues. The present 24 h urine measurements cannot distinguish direct removal from intracellular stores from capture of circulating or extracellular cadmium, or from cadmium entering extracellular fluid through ongoing tissue exchange. Total-element ICP-MS likewise does not directly identify Cd-DTPA.

Safety is an additional important consideration, particularly because cadmium can damage the kidneys and Ca-DTPA may also increase the loss of essential metals through urine. Studies in healthy volunteers have shown increased urinary excretion of zinc and manganese, along with transient changes in the blood levels of essential metals after DTPA administration [7]. The Ca-DTPA prescribing information also warns about the risk of trace-metal depletion with repeated treatment [10]. For approved radionuclide indications, the prescribing information recommends switching to Zn-DTPA after the initial Ca-DTPA dose when further treatment is needed, together with appropriate monitoring and supplementation [10]. However, these recommendations do not establish a treatment regimen for cadmium poisoning. Animal studies have explored whether zinc supplementation might help reduce some of these effects [25], but the findings are not enough to recommend a specific supplementation regimen for patients with cadmium exposure. Zinc supplementation during Ca-DTPA treatment should be distinguished from administration of Zn-DTPA, as these are different interventions. These concerns highlight the importance of monitoring kidney function and essential trace elements, especially if repeated Ca-DTPA treatment is being considered.

Our study was not designed to determine whether Ca-DTPA is safe or whether its potential benefits in removing cadmium outweigh the risks. We did not systematically assess kidney injury, the loss of essential metals, adverse effects, or long-term clinical outcomes. Although we observed increased urinary cadmium excretion after treatment, this finding alone does not justify repeated Ca-DTPA administration in patients with cadmium poisoning.

Post-chelation urinary cadmium measurements should not be interpreted using reference ranges established for unprovoked urine samples, nor should they be used independently to diagnose cadmium poisoning. Chelator administration changes urinary excretion, and validated diagnostic thresholds for post-chelation cadmium measurements have not been established. The present paired measurements address the narrower pharmacodynamic question of whether urinary cadmium changes after a defined treatment episode.

Paired 24 h urine measurements can address that limited within-person pharmacodynamic question, provided collection timing and completeness remain carefully documented. Serial timed urine collections would better characterize the duration of the excretion response, and appropriately controlled fecal measurements could assess nonrenal elimination; work in radionuclide decorporation demonstrates why a single first-day urine interval may not capture total post-treatment elimination [26]. Cadmium-specific studies would also need to account for dietary cadmium in feces.

This study has several limitations. It is retrospective, single-center, and based on a selected clinical population treated for suspected gadolinium-related conditions rather than cadmium poisoning. Selection depended on the availability of paired laboratory measurements. We could not independently verify collection completeness, and the post-treatment interval began after the first post-dose void. The study had no untreated or placebo control group, and concomitant medications, hydration, exposure variability, and other clinical factors could not be separated from treatment-associated change. The study did not systematically assess blood cadmium, renal tubular injury biomarkers, tissue measurements, serial post-treatment kinetics, fecal elimination, or cadmium-specific clinical outcomes. Essential-metal losses and long-term safety were not primary endpoints. These limitations preclude conclusions about therapeutic efficacy, sustained tissue depletion, or clinical benefit.

Future studies should focus on patients with documented cadmium exposure and include clinical outcomes that can help determine whether treatment provides any real benefit. Ideally, these studies should be prospective, with clearly defined treatment and control groups. Repeated blood and urine cadmium measurements would help us better understand how cadmium levels change over time, while kidney function tests and markers of renal tubular injury would provide important information about safety. Bone health and mineral metabolism should also be assessed when relevant, along with possible losses of essential trace elements. Collecting urine at several time points, and fecal samples where feasible, would give a more complete picture of cadmium elimination. Clinical outcomes should be assessed using standardized methods. Finally, studies directly comparing different chelators would be needed before we could determine whether one treatment offers an advantage over another.

## 4. Conclusions

In this retrospective study of 60 patients, urinary cadmium excretion increased significantly after intravenous Ca-DTPA administration. The mean 24 h urinary cadmium excretion rose from 0.32 to 1.31 µg/24 h, corresponding to a 4.09-fold increase in the group mean. The median increase for individual patients was approximately 3.7-fold (IQR, 2.4–5.8), and all 60 patients showed an increase after treatment (Wilcoxon signed-rank *p* < 0.001; exact sign-test *p* = 1.73 × 10^−18^).

These data suggest that Ca-DTPA can mobilize cadmium that is accessible to the chelator. However, they do not tell us whether patients had cadmium poisoning, how much cadmium was actually removed from the body’s stores, or whether this removal was sustained. We also cannot draw conclusions about the safety of repeated treatment or any potential clinical benefit. Further controlled studies are needed to better understand cadmium elimination after Ca-DTPA and whether this approach could help treat cadmium-related disease.

## Figures and Tables

**Table 1:** Participant characteristics and treatment/collection protocol.

Characteristic	Value
Participants, n	60
Women, n (%)	30 (50%)
Men, n (%)	30 (50%)
Age, years, mean (range)	49 (21–79)
Clinical indication	Suspected gadolinium-related condition; not recruited for cadmium poisoning
Ca-DTPA dose	1 g intravenous
Baseline urine collection	24 h urine collection before Ca-DTPA administration
Post-treatment urine collection	24 h collection beginning after the first post-treatment void, generally approximately 1 h after Ca-DTPA administration

**Table 2:** Quantitative paired urinary cadmium outcomes.

Outcome	Summary Statistic	Value
Pretreatment urinary Cd, µg/24 h	Mean (range)	0.32 (0.03–0.99)
Post-treatment urinary Cd, µg/24 h	Mean	1.31
Mean paired increase in urinary Cd, µg/24 h	Mean (95% CI)	0.99 (0.75–1.23)
Ratio of group means	1.31/0.32	4.09
Individual post/pre ratio	Median (IQR)	≈3.7 (2.4–5.8)
Individual post/pre ratio	Range	1.2–9.5
Participants with increased Cd excretion	n/N (%)	60/60 (100%)
Paired comparison	Wilcoxon signed-rank, two-sided	*p* < 0.001
Sensitivity analysis	Exact sign test, two-sided	*p* = 1.73 × 10^−18^

Abbreviations: Cd, cadmium; IQR, interquartile range. The ratio of group means was calculated by dividing mean post-treatment urinary cadmium excretion by the corresponding pretreatment mean. Individual post/pre ratios were calculated separately for each participant. All statistical comparisons were paired and two-sided.

## Data Availability

The original contributions presented in this study are included in the article. Further inquiries can be directed to the corresponding author.
